# Association between Salivary Immune Markers and Oral Health Conditions in Pregnant Women

**DOI:** 10.21203/rs.3.rs-5968375/v1

**Published:** 2025-04-14

**Authors:** Nora Alomeir, Xinyue Mao, Ruqian Yang, Nasser Assery, Sally Quataert, Antti Seppo, Xingyi Lu, Tong Tong Wu, Jin Xiao

**Affiliations:** Eastman Institute for Oral Health; University of Rochester Medical Center; Eastman Institute for Oral Health; Eastman Institute for Oral Health; University of Rochester Medical Center; University of Rochester Medical Center; Eastman Institute for Oral Health; University of Rochester Medical Center; Eastman Institute for Oral Health

**Keywords:** Caries, Streptococcus mutans, Candida albicans, Immunity, Candidiasis, Pregnancy

## Abstract

**Objectives:**

This study aims to assess levels of salivary immune markers in women during the 3rd trimester of pregnancy and explores the association between immune markers and dental caries, as well as oral carriage of oral pathogens *Candida albicans* and *Streptococcus mutans.* These organisms are known to be associated with oral infectious diseases such as dental caries and oral candida infection.

**Methods:**

Pregnant women from underserved background participated in this study (n = 181). Participants received a comprehensive oral examination by trained and calibrated dentists. Levels of 36 immune markers in unstimulated saliva samples were measured using Bio-Plex200 (Luminex). Salivary *S. mutans* and *C. albicans* were quantified using viable count (CFU/ml). Quantities of immune markers and colony-forming-units of *S. mutans* and *C. albicans* were converted to natural log data for statistical analysis. Latent class analysis was used to assess the clustering effect of immune markers among the participants.

**Results:**

The study found high quantities of salivary immune markers in pregnant women with varying detection rates. Univariate analysis found a higher salivary level of IFN-g and TNF-b among women with < 4 decayed teeth, compared to those with ≥ 4 decayed teeth (p < 0.05). Among women with more than 10^5^ CFU/ml *S. mutans* in saliva, FLT-3L, IL-17a, TNF-B, and VEGF-a levels were significantly decreased (p < 0.05), while G-CSF levels were significantly increased (p < 0.05). Lower levels of VEGF-a, Eotaxin and IL-9 were found among the individuals with salivary *C. albicans* (p < 0.05). Two distinct clusters were identified among the 181 participants, low-level and high-level of immune markers. The logistic regression model with Least Absolute Shrinkage and Selection Operator (LASSO) penalty identified several potential variables associated with high-level of salivary immune markers. Debiased results indicated statistically significant association between plaque *C. albicans* and *S. mutans* and immune markers (p < 0.05), with higher levels of these variables correlating with lower level of immune markers. In summary, our research underscores the intricate relationship between caries status, microbial populations, and immune markers in pregnant women.

## 1. Introduction

Pregnancy is a natural physiological process that involves temporary alterations in a woman's bodily structure, hormonal balance, metabolism, and immune function [1]. In mothers, inadequate and untreated prenatal oral health care significantly contributes to poor maternal oral health, the potential for negative birth outcomes, and a heightened risk of early childhood caries in their offspring [2]. Dental caries is the most common and widespread noncommunicable disease impacting people globally today[3]. In terms of immunological response, both periodontitis and caries exhibit a similar pattern where the nuclear factor kappa B (NF-kB) is activated upon bacterial challenge, which then triggers the release of pro-inflammatory cytokines [4]. Caries triggers oral immune responses through the release of the cytokines interleukin 6 (IL-6), IL-1, IL-8, as well as tumor necrosis factor (TNF)-α [5, 6].

In early childhood caries (ECC), a significant global health concern, the roles of *Streptococcus mutans* and *Candida albicans* are critically examined, particularly focusing on their synergistic interactions which potentiate cariogenicity [7]. *S. mutans,* known for its acidogenic and biofilm-forming capabilities, significantly contributes to tooth demineralization by metabolizing carbohydrates into acids. Similarly, *C. albicans* enhances the cariogenic environment through acid production and its ability to form complex biofilms on tooth surfaces that can encapsulate *S. mutans*. Their interaction in the oral cavity reveals cooperative dynamics; synergistically, they enhance each other's cariogenic effects by metabolic cooperation and increased biofilm viability, resilience, and complexity [8]. Understanding these microbial interactions provides crucial insights into the pathogenesis of ECC and offers potential therapeutic targets to disrupt these interactions and reduce caries prevalence and severity [8].

Previous studies explored the maternal influence of *C. albicans* and *S. mutans* transmission in early life, highlighting the importance of women’s oral health during pregnancy [9, 10]. This study aimed to assess levels of salivary immune markers (cytokines, chemokines, and growth factors) in women during the 3rd trimester of pregnancy and explore the association between immune markers and dental caries, as well as oral carriage of *C. albicans* and *S. mutans*. Thus, we hypothesize that elevated levels of specific immune markers correlate with varied loads of cariogenic microorganisms during pregnancy.

## 2. Materials and Methods

### 2.1. Study Population

This study utilized data and saliva samples from a cohort study involving 186 pregnant women who were selected from economically disadvantaged demographics at the University of Rochester Medical Center, specifically from Highland Family Medicine (HFM) and Eastman Institute for Oral Health (EIOH) during the years 2017 to 2020 [10, 11]. Of these women, 181 completed the required saliva sample collections and were included in this study. All participants provided written informed consent. The study's protocol was approved by the University of Rochester Research Subject Review Board, under approval number #1248 [12]. All methods were performed in accordance with the relevant guidelines and regulations. The study cohort consisted of pregnant women who met specific inclusion and exclusion criteria. The inclusion criteria required participants to be over 18 years old, in their third trimester of pregnancy, carrying a single fetus, and eligible for Medicaid insurance, indicating an income below 138% of the Federal Poverty Line. The exclusion criteria ruled out participants with severe systemic diseases, oral cancer, cleft lip or palate, and those who had used antifungal medication in the past three months [13]. These criteria ensured the selection of a study population suitable for our research objectives.

### 2.2. Data Collection and Examination Methods

The examination and data collection methods used in this study involved comprehensive oral examinations and detailed data/sample collection processes. For instance, oral examinations were conducted during the mother’s third trimester. These examinations assessed caries scores, plaque index, and oral candidiasis, and involved the collection of oral samples such as saliva, plaque, and mucosal swabs, which were performed by 1 of 3 calibrated dentists using standard dental equipment and portable lighting [10, 11]. The supragingival plaque was collected using a sterile scaler and immersed in an Eppendorf tube containing 1 ml of saline solution. A homogeneous mixture was created by sonication and then plated on appropriate agar plates for colony identification. Medical background data, including physician-diagnosed systemic diseases, medication use, and smoking status, were gathered through self-reporting and confirmed using electronic medical records [11]. Demographic, socioeconomic, and oral hygiene practices data were collected via questionnaires. Saliva samples were collected by instructing participants to abstain from eating, drinking, or brushing their teeth for 2 hours prior to collection, ensuring the samples were transported to the lab within 2 hours and stored appropriately for further analysis [13].

### 2.3. Colonies Identification

Salivary and plaque *S. mutans* and *C. albicans* were quantified using viable count (CFU/mL) [14]. The saliva and plaque samples were processed within 2 hours after collection by plating them on BBL^™^ CHROMagar^™^ Candida (BD, Sparks, MD, USA) using an automated EddyJet Spiral Plater (IUL, SA, Barcelona, Spain) and then incubated at 37°C for 48 hours to isolate *C. albicans*. This medium allows for the preliminary identification of various significant *Candida* species, including *C. albicans*, by observing the color and morphology of the colonies [13]. The samples were also plated on Mitis Salivarius agar with Bacitracin, incubated at 37°C for 48 hours, to isolate *S. mutans*, which were identified based on their colony morphology [15]. *C. albicans* and *S. mutans* were further verified using colony polymerase chain reaction method [16]. Colony-forming unit (CFU) counting methods were employed for the quantification of microbial species.

### 2.4. Immune Markers Measurement

Thawed unstimulated saliva samples were subjected to high-speed ultracentrifugation to remove large debris [17]. The profiles of host immunological markers were analyzed using a MILLIPLEX^®^ Human Cytokine/Chemokine/Growth Factor Panel A - Immunology Multiplex Assay, Cat# HCYTA-60K (Merck Millipore, Darmstadt, Germany) [18]. A total of 36 analytes were assessed, encompassing a broad spectrum of immune biomarkers found in saliva. These included epidermal growth factor (EGF), fibroblast growth factor 2 (FGF2), C-C motif chemokine ligand 11 (CCL11, formerly eotaxin), transforming growth factor alpha (TGFA), colony stimulating factor 3 (CSF3, formerly granulocyte colony stimulating factor), fms related receptor tyrosine kinase 3 ligand (FLT3LG), vascular endothelial growth factor A (VEGFA), C-X3-C motif chemokine ligand 1 (CX3CL1, formerly fractalkine), C-X-C motif chemokine ligand 1 pseudogene 1 (CXCL1P1, formerly growth-regulated oncogene), and additional markers such as interleukin 1 receptor antagonist (IL-1RN), interleukin 4 (IL-4), interleukin 10 (IL-10), interleukin 8 (IL-8), interleukin 18 (IL-18), interleukin 1 alpha (IL-1a), interleukin 1 beta (IL-1b), interleukin 2 (IL-2), interleukin 6 (IL-6), interleukin 12p40 (IL-12p40), interleukin 12p70 (IL-12p70), interleukin 17a (IL-17a), tumor necrosis factor alpha (TNF-a), tumor necrosis factor beta (TNF-b), monocyte chemoattractant protein-1 (MCP-1), monocyte chemoattractant protein-3 (MCP-3), macrophage-derived chemokine (MDC), macrophage inflammatory protein-1 beta (MIP-1b), regulated on activation, normal T cell expressed and secreted (RANTES), soluble CD40 ligand (sCD40L), interferon alpha 2 (IFN-a2), interferon gamma (IFN-g), interleukin 5 (IL-5), interleukin 9 (IL-9), interleukin 13 (IL-13), interleukin 15 (IL-15), Interferon gamma-induced Protein 10 (IP-10), reflecting a diverse range of immune responses in the oral environment.

### 2.5. Statistical Analysis

Quantities of immune markers and colony-forming-units of *S. mutans* and *C. albicans* were converted to natural log data to normalize their distribution. The R software version 4.2.1 (R Foundation for Statistical Computing, Vienna, Austria) was utilized for all computations. Univariate analysis highlighted individual marker characteristics. Latent Profile Analysis then clustered subjects based on these markers, revealing distinct immune response patterns. Further univariate analysis compared these clusters, uncovering significant differences in marker levels, Chi squared test was utilized for binary variables while an unpaired t-test was used for continuous variables. L1-penalized Logistic Regression was applied to explore the relationship between clusters and various covariates, enhancing model accuracy and interpretability. Post-selection inference provided reliable p-values, ensuring robust association tests between clusters and covariates, thus offering a comprehensive understanding of the characteristics’ impact on immune markers.

## 3. Results

181 pregnant women completed in this study. Among the participants, 70% were ≤ 30 years old, 58% had either middle school or high school education level, and 78% were unmarried. Regarding the participants’ race, 53% were Black, 29.3% were White, and 17.7% were from other racial backgrounds.

### 3.1. Univariate Analysis of Individual Immune Markers

In this study, we analyzed the detection rates and concentrations of various immune markers in 181 pregnant women. The immune markers evaluated showed varying levels of detectability and concentrations (Table 1). Markers such as EGF, IL-1a, IL-1ra, IL-8, IL-18, MCP-1, IL-1B, and IL-6 had high detection rates of over 90%, with IL-1a, IL-1ra, and EGF being detected in all participants. The mean concentrations of these markers varied widely, with IL-1ra and IL-1a showing notably high mean values of 2009.6 ± 2027.7 pg/mL and 1870.4 ± 2298 pg/mL, respectively. Other markers, such as TGF-a, Eotaxin-1, and CD40L, had moderate detection rates ranging from 70–87%, with corresponding mean concentrations of 7.7 ± 7 pg/mL, 14.6 ± 13.1 pg/mL, and 77.4 ± 88.8 pg/mL. Notably, several markers including IL-4, IL-5, and IL-2 had low detection rates of 20–33%, reflecting lower overall presence in the cohort. The median concentrations and ranges also varied significantly, with markers like IL-1ra and EGF exhibiting wide concentration ranges (55.8–13147.7 pg/mL and 28.5–4789.4 pg/mL, respectively). These findings illustrate the diverse immune profiles among pregnant women, highlighting both prevalent and less common immune markers in this population.

**Figure 1:**
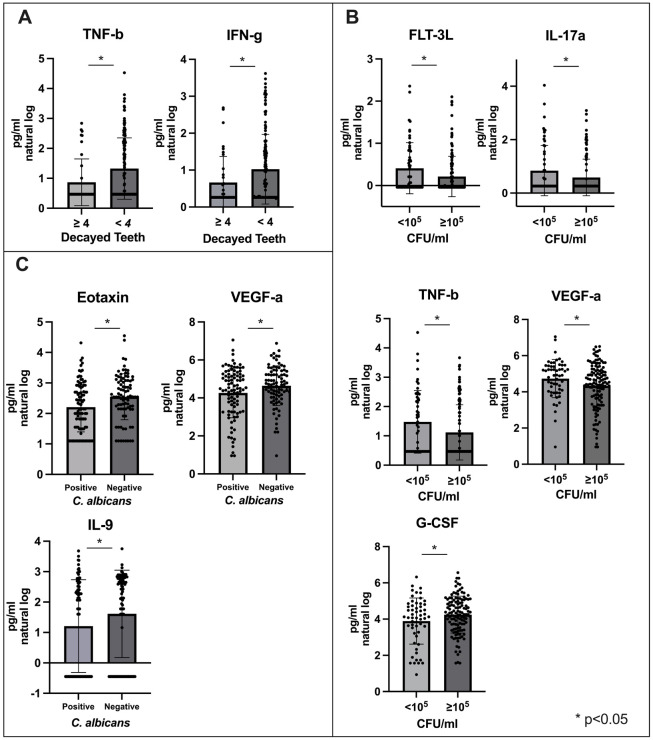
The results indicate significant differences in immune marker levels under various conditions. (A) reveals that levels of TNF-b and IFN-g are significantly higher in individuals with fewer than 4 decayed teeth compared to those with 4 or more decayed teeth. (B) Shows that levels of FLT-3L, IL-17a, TNF-b, and VEGF-a are significantly higher in samples with CFU/ml < 10^5 compared to those with CFU/ml ≥ 10^5, while G-CSF levels are significantly lower in samples with CFU/ml < 10^5 compared to those with CFU/ml ≥ 10^5. Figure (c) demonstrates that levels of Eotaxin, VEGF-a, and IL-9 are significantly higher in C. albicans positive samples compared to negative ones.

Pregnant women with ≥ 4 decayed teeth had lower salivary level of IFN-g and TNF-b, compared to those with < 4 decayed teeth (p < 0.05) (Fig. 1A). Among women with more than 10^5^ CFU/ml *S. mutans* in saliva, FLT-3L, IL-17a, TNF-B, and VEGF-a levels were significantly decreased (p < 0.05), while G-CSF levels were significantly increased (p < 0.05) (Fig. 1B). A lower level of Eotaxin, VEGF-a and IL-9 was found among the individuals with salivary *C. albicans* (p < 0.05) (Fig. 1C). The findings underscore the distinct immune responses associated with dental decay, microbial load, and fungal presence, highlighting their potential role as biomarkers for oral health and infection.

### 3.2. Immune Markers Levels and Clusters of Saliva During Pregnancy

**Figure 2:**
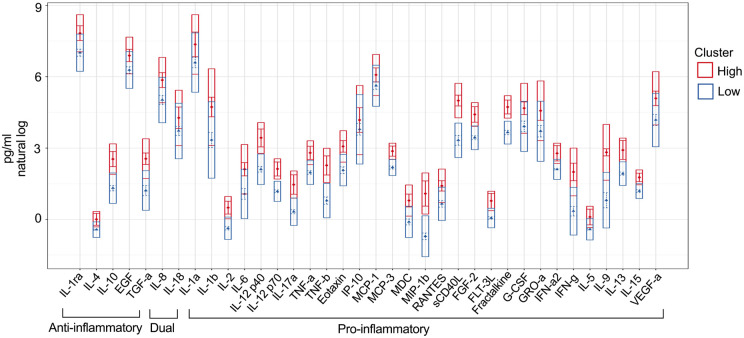
Based on salivary immune markers, two clusters of pregnant women were generated using a latent model. Two distinct clusters emerged from 181 samples: one with low immune marker levels (n=127) and one with high immune marker levels (n=54). Despite the minimal overlap between these two clusters, the classification effectively differentiates between clusters based on immune marker levels.

In order to study 36 immune markers simultaneously, we conducted the Latent Profile Analysis (LPA) using the natural log-transformed value of all immune markers to generate clusters and analyze variations of covariates between clusters. As most of the popular clustering methods, LPA requires pre-specified number of clusters. Among the study population, two clusters were identified, cluster with higher immune markers levels (Cluster high, n = 54) and cluster with lower immune markers levels (Cluster low, n = 127) (Fig. 2). Moreover, immune markers were organized based on function as anti-inflammatory, dual function, and pro-inflammatory markers.

The comparison between the Low (n = 127) and High (n = 54) groups across various binary variables revealed several significant findings (Table 2). In terms of demographics, there were no significant differences observed in age > 30 (p = 0.148), Black race (p = 0.710), or Hispanic ethnicity (p = 1). Medical record variables, including yeast infection, antibiotic use, antifungal use, prenatal inhaler use, prenatal diabetes, prenatal asthma, prenatal emotional condition, depression score > 12, prenatal hypertension, and prenatal smoking, did not show significant differences between the groups (p > 0.05). In socioeconomic status, while employment status was similar between the groups (p = 1), marital status was significantly higher in the High group (33.33%) compared to the Low group (17.32%) (p < 0.05). Regarding birth outcomes, the incidence of low birth weight was higher in the High group (7.41%) compared to the Low group (2.36%), though this difference was not statistically significant (p = 0.234). Oral health variables displayed notable differences: the detection of Saliva *Candida* (p < 0.05), Saliva *S. mutans* (p < 0.05), Plaque *Candida* (p < 0.001), and Plaque *S. mutans* (p < 0.001) was significantly lower in the High group compared to the Low group, however, brushing twice daily did not show any significant difference. These results highlight that marital status and specific oral health indicators significantly differ between the Low and High groups, while other demographic, medical, socioeconomic, and birth outcome variables do not.

The comparison of various continuous variables between the Low (n = 127) and High (n = 54) groups revealed significant differences in several hormonal levels and microbial counts (Table 3). The mean age, gestational week and infant birth weight were similar between the two groups, with no significant differences observed. Dental health indicators such as DMFT, DMFS, and ICDAS scores also did not differ significantly. However, hormonal analysis showed significantly higher mean levels of cortisol (p < 0.0001), estradiol (p < 0.001), testosterone (p < 0.001), T3 (p < 0.001), and T4 (p < 0.05) in the Low group compared to the High group. Progesterone levels, though higher in the Low group, did not reach statistical significance (p = 0.133). Microbial analysis revealed significantly higher counts of *C. albicans* in both saliva (p < 0.05) and plaque (p < 0.0001) in the Low group. Additionally, the plaque counts of S. mutans were significantly higher in the Low group (p < 0.05), while saliva counts of S. mutans did not differ significantly. These findings suggest notable associations between the measured variables and the Low and High groups, particularly in the context of hormonal and microbial differences, warranting further investigation.

### 3.3. Variables Associated with Immune Markers Cluster

The logistic regression model with LASSO penalty identified several variables significantly associated with high immune markers, using low immune markers as the reference (Table 4). Positive coefficients were observed for being married (0.196), low birth weight (0.109), and prenatal diabetes (0.160), suggesting that these factors are associated with a higher likelihood of having high immune markers. Conversely, negative coefficients were observed for cortisol (−0.180), testosterone (−0.065), plaque *C. albicans* (−0.574), and plaque *S. mutans* (−0.763), indicating that higher levels of these variables are associated with a decreased likelihood of having high immune markers. These results highlight the complex interplay between various demographic, hormonal, and microbial factors in determining immune marker levels, emphasizing the need for a multifactorial approach in understanding and addressing the underlying mechanisms influencing immune status. Since LASSO penalty shrank the estimator toward zero, the coefficients were biased. To make inference, we applied the debiased procedure (Table 5) [19]. The debiased result is as follows: Two plaque-related variables (Plaque *C. albicans* and Plaque *S. mutans*) had p-values less than 0.05, indicating a statistically significant effect on the likelihood of having high immune markers. The negative debiased coefficients for these plaque-related variables suggest that higher levels of these variables are associated with a lower proportion of subjects having high immune markers.

**Figure 3:**
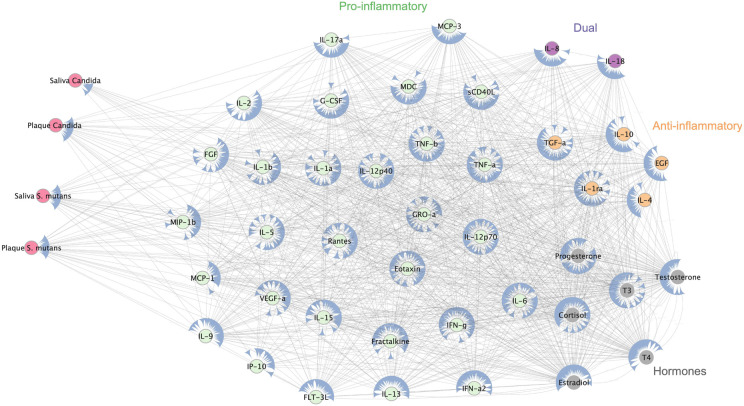
This network illustrates the relationships between *Candida* and *S. mutans*levels (pink nodes) in saliva and plaque and various immune and hormonal markers. Immune markers are categorized as pro-inflammatory (green), anti-inflammatory (orange), and dual-function (purple), revealing their potential roles in microbial-host interactions.

Cytoscape software was utilized to construct and analyze the complex interactions between pathogens, immune markers and hormones [20]. The network analysis uncovered distinct associations between salivary and plaque levels of *Candida* and *S. mutans* with a diverse array of immune markers and hormones (Fig. 3). *Candida* exhibited strong correlations with both pro-inflammatory markers, including IL-1β, TNF-α, and IL-17A, as well as anti-inflammatory markers such as IL-10 and TGF-α. This suggests that Candida plays a role in modulating a wide-ranging inflammatory response. In contrast, S. mutans, particularly in saliva, was primarily linked to pro-inflammatory cytokines like MCP-1, IL-12p40, and RANTES, indicating a more focused inflammatory activation. Additionally, hormones markers such as cortisol and progesterone were associated with both microbial species, implying systemic regulatory influences that may mediate the immune response to these microbes. Collectively, these findings emphasize the intricate interplay between oral microbiota, immune pathways, and systemic hormonal regulation, highlighting potential biomarkers for microbial-driven inflammation and overall systemic health.

## 4. Discussion

The feasibility of using salivary immune markers to monitor physiological changes during pregnancy and their impact on oral health, particularly dental caries, is supported by recent advancements in non-invasive diagnostic methods. Saliva collection is advantageous due to its non-invasive nature, lower biosafety requirements, and ease of repeated sampling, making it ideal for longitudinal studies in pregnant women [21].Our study provides valuable insights into the immune changes that occur during the third trimester of pregnancy, focusing on salivary immune markers and their associations with hormonal levels and microbial counts. High detection rates (> 90%) were observed for markers such as EGF, IL-1a, IL-1ra, IL-8, IL-18, MCP-1, IL-1B, and IL-6, with IL-1ra and IL-1a showing notably high mean concentrations of 2009.6 ± 2027.7 pg/mL and 1870.4 ± 2298 pg/mL, respectively. Markers like TGF-a, Eotaxin-1, and CD40L had moderate detection rates (70%-87%), while IL-4, IL-5, and IL-2 had low detection rates (20%-33%). Significant associations were found between oral health indicators and immune responses, with lower salivary levels of IFN-g and TNF-b in women with ≥ 4 decayed teeth and variations in immune marker levels in relation to microbial loads and fungal presence. Latent Profile Analysis identified two clusters of immune marker levels, highlighting significant differences in demographic, socioeconomic, hormonal, and microbial variables, with higher cortisol, estradiol, testosterone, T3, and T4 levels and higher counts of *C. albicans* and *S. mutans* in the low-level cluster. Logistic regression with LASSO penalty revealed significant associations between marital status, low birth weight, diabetes, hormonal levels, and microbial counts with immune marker levels, underscoring the complex interplay of factors influencing immune status during pregnancy.

### 4.1. The Rationale Behind Immune Markers Selection

The rationale for selecting this panel of 36 immune markers is based on current literature indicating multiple immune markers associated with caries. However, in our study, we were unable to include all immune markers mentioned in literature due to constraints in sample volume and the cross-reactivity of several markers when using the same assay kit. A study by Baker et al. examined 38 immune markers among children (EGF, FGF2, CCL11(eotaxin), TGFA, CSF3, CSF2, FLT3LG, VEGFA ,CX3CL1 (fractalkine), CXCL1P1, CCL7, CCL22, CXCL8, CXCL10(IP-10), CCL2, CCL3, CCL4, IFNA2, IFNG, IL-1a, IL-1b, IL-1RN, IL-2, IL-3, IL-4, IL-5, IL-6, IL-7, IL-9, IL-10, IL-12p40, IL-12p70, IL-13, IL-15, IL-17, CD40L, TNF, and LTA) finding that ten were elevated due to dental caries [17]. These markers include epidermal growth factor (EGF), interleukin 10 (IL-10), colony stimulating factor 3 (CSF3), interleukin 1 receptor antagonist (IL-1RN), colony stimulating factor 2 (CSF2), CCL22, interleukin 13 (IL-13), interleukin 15 (IL-15), and interleukin 6 (IL-6), all of which have significant roles in the development and progression of dental caries [17]. Another cross-sectional study examined 12 immune markers (IFN-γ, IL-1α, IL-1β, IL-4, IL-5, IL-6, IL-8, IL-10, IL-13, IP-10, TNF-α, VEGF-A), the concentrations of IL-1β, IL-6, IL-8, and IL-10 were markedly elevated among the caries group [22]. FGF expression plays a crucial role in various stages of tooth development, including the initiation of teeth and the formation of mineralized tissues. In rodents, FGFs are uniquely vital for maintaining the stem cell niche that supports the continuous growth of their incisors throughout their lives [23]. Moreover, previous studies have identified elevated levels of various cytokines in caries-affected dental pulp and/or odontoblasts. These include transforming growth factor-β1 (TGFβ1), vascular endothelial growth factor (VEGF), C-C chemokine ligand 2 (CCL2/MCP1), CCL20/MIP3α, interleukin 8 (IL8/CXCL8), CXC chemokine ligand 10 (CXCL10), epithelial cell-derived neutrophil attractant 78 (ENA78), IL-1β, IL2, IL4, IL6, IL10, IL11, interferon-γ (IFN-γ), and tumor necrosis factor-α (TNF-α) [24].

In adults, the selected markers include TNFα, IFN-g, GM-CSF, IL-2, IL-4, IL-6, IL-8, and IL-10, reflecting the complex interactions between the immune system and cariogenic factors in adult populations [25]. This comprehensive panel aims to cover a broad spectrum of immune responses to provide a thorough understanding of caries-related immunological mechanisms. Furthermore, multiple studies indicated the role of IL-17A in protection against *C. albicans* (an important cariogenic organism) and its regulation of antifungal immunity [26], IL-17A is crucial for combating candidiasis both at mucosal and systemic levels [27]. A single arm clinical trial on adult participants with candidiasis also revealed significant reduction in the levels of salivary cytokines Eotaxin and Fractalkine in the group receiving an antifungal intervention [28].

### 4.2. The Effect of Salivary Hormones on the Immune Markers

According to Mor and Cardenas (2010), the third trimester is characterized by a renewed pro-inflammatory state, essential for initiating labor and ensuring successful delivery. This phase involves elevated levels of pro-inflammatory cytokines such as TNF-α, IL-1, and IL-6, which are crucial for processes like cervical ripening, membrane rupture, and uterine contractions [29]. Our analysis revealed that certain salivary hormones and microbial counts are significantly associated with immune marker levels during this critical period. Specifically, higher levels of cortisol and testosterone were negatively associated with high immune markers. Cortisol functions as an immunosuppressant by downregulating key inflammatory transcription factors such as NF-kB and AP-1, while simultaneously upregulating suppressor of cytokines (SOCS). This upregulation of SOCS inhibits STAT phosphorylation, leading to a reduction in downstream pro-inflammatory gene transcription, thereby weakening the pro-inflammatory response [30, 31]. The observed negative association between cortisol and high immune markers is a significant finding that aligns with the well-documented immunosuppressive effects of cortisol. Cortisol, a glucocorticoid hormone, plays a crucial role in the body’s stress response and is known to suppress various components of the immune system, including cytokine production, antibody formation, and the activity of immune cells such as T-lymphocytes and macrophages[32]. This immunosuppressive effect can reduce the body's ability to mount an effective immune response, which may explain why higher cortisol levels are associated with lower immune markers. Testosterone similarly exhibits immunosuppressive effects by inhibiting the production of pro-inflammatory cytokines and reducing the activity of various antimicrobial immune cells, contributing to the reduced immune marker levels [33].

The results of this study are consistent with previous research, for example a study by Yang et al. (2024), which identified significant associations between salivary hormones, dental caries, and cariogenic microorganisms during pregnancy [13]. Yang et al. reported that higher levels of salivary progesterone, estradiol, testosterone, and cortisol were positively associated with increased carriage of S. mutans and higher caries risk. Our study corroborates these findings, emphasizing the role of salivary hormones in modulating oral health and immune markers.

### 4.3. The Influence of Marital Status and Diabetes on the Immune Response

Conversely, demographic factors such as being married and having diabetes were positively associated with high immune markers. The social support associated with being married may help mitigate stress and its associated immunosuppressive effects, thereby contributing to higher immune markers. Additionally, the elevated immune markers observed in married pregnant women may reflect an enhanced ability to cope with these physiological demands due to reduced stress levels [34]. The third trimester's pro-inflammatory state, while essential for labor, can also increase susceptibility to infections and inflammatory conditions [34]. Thus, the supportive environment provided by a stable marital relationship might play a crucial role in balancing the immune response, potentially reducing the risk of adverse outcomes related to excessive inflammation. Furthermore, the relationship between diabetes and altered immune marker levels during pregnancy highlights the complex interplay between metabolic and immune systems [35]. Pregnant women with diabetes, particularly gestational diabetes mellitus (GDM) and type 1 diabetes mellitus (T1DM), demonstrate a distinctive immune profile characterized by heightened inflammation. This pro-inflammatory state is evidenced by elevated levels of biomarkers such as CRP, IL-6, and PAI-1 [35]. Hence, diabetes was positively correlated with high immune marker levels in our study cohort. Previous studies have shown that individuals with diabetes mellitus (DM) tend to have a higher incidence of both coronal and root caries, particularly when their glycemic control is inadequate [36]. Additionally, DM is linked to changes in the immune response, which may affect salivary cytokine levels regardless of the presence of *C. albicans* or *S. mutans.*

### 4.4. Immune Markers and Low Birth Weight: Potential Predictive Biomarkers

A paper by Padilla-Cáceres et al. examined the relationship between periodontal disease and adverse pregnancy outcomes, such as preterm birth and low birth weight. The study conducted a comprehensive review of systematic reviews and meta-analyses to explore this association. The findings indicated that periodontal disease in pregnant women is associated with an increased risk of preterm birth and low birth weight in newborns [37]. This association may be due to inflammatory processes or the translocation of periodontal pathogens. This explains the relationship between poor oral health and low birth weight. An unexpected finding was evaluated in our study indicating a positive relationship between high immune markers and low birth weight, this suggests that salivary immune status in pregnant women may serve as a predictive biomarker for adverse birth outcomes, such as low birth weight. However, this potential application requires further validation through future studies to confirm its accuracy and reliability.

### 4.5. Immune Markers in Saliva as Indicators of Caries and Microbial Imbalance

Certain cytokines and chemokines have shown promise as biomarkers for caries. These immune markers are part of the body's response to inflammation and infection, and their presence in saliva can reflect the underlying pathological processes leading to caries formation [38]. The number of decayed surfaces (DS) showed a trend towards significance (p = 0.055), suggesting women with high immune markers levels have less teeth surfaces affected by caries (Table 3). Also, the number of filled surfaces (FS) shows borderline significance (p = 0.053), indicating women with high immune markers levels have less restored teeth surfaces suggesting elevated immune markers showing a protective mechanism by reducing the need for restorative treatment. Microbial analysis highlighted that higher counts of plaque *C. albicans* and plaque *S. mutans* were negatively associated with high immune markers. The significantly lower *S. mutans* count in dental plaque among individuals with high immune markers, despite no difference in salivary levels, suggests that host immune responses may locally modulate bacterial colonization at the site of caries initiation. This supports the ecological plaque hypothesis and highlights the potential role of immunity in maintaining oral microbial balance[39].

This suggests that an altered oral microbiome, potentially facilitated by a suppressed immune system due to higher cortisol and testosterone levels, can influence immune marker levels. The presence of these cariogenic microorganisms may reflect an imbalance in the oral environment due to hormonal changes and immune modulation during pregnancy.

### 4.6. Study Limitations

The limitations of this study include the inherent constraints of a cross-sectional design, which preclude the determination of causality between salivary immune markers and the dental caries status or presence of cariogenic pathogens. Additionally, this study faced constraints in sample volume and did not account for all potential confounding variables such as dietary habits, detailed salivary characteristics, and the impact of diurnal fluctuations on immune markers. The cross-reactivity of several markers when using the same assay affected the immune markers selection process, limiting other important immune markers from being included in the analysis. The study's participant pool was drawn exclusively from underserved communities in Upstate New York, limiting the generalizability of the findings to other populations or regions. Future research should consider longitudinal studies with more diverse populations and geographical settings to further elucidate the role of immune markers in oral health during pregnancy. Additionally, understanding the maternal influence on immunity in early life and its association with cariogenic microorganisms, as well as assessing the underlying mechanisms that link immune markers with caries risk and adverse birth outcomes, will be crucial.

## 5. Conclusions

In conclusion, our study highlights the complex interplay between hormonal levels, microbial counts, and immune markers in pregnant women. These findings suggest that monitoring salivary hormones and microbial presence could provide valuable insights into immune status and potential risks for adverse oral health outcomes during pregnancy. Overall, 36 immune markers were observed in saliva with varying detection rates among pregnant women from low-income and minoritized background, indicating the feasibility of using saliva as a diagnostic sample. Further research is warranted to comprehensively assess the role of salivary immune markers in understanding the immunological mechanisms between oral microorganisms and the host, and to develop targeted interventions to improve maternal and fetal health.

## Figures and Tables

**Table 1:** Immune markers detection rate and levels in 181 pregnant women

Immune marker	Detection rate	Mean (pg/mL) ± SD	Median (pg/mL)	Range (pg/mL)
**EGF**	100%	879.4 ± 777.8	641.2	28.5 _ 4789.4
**IL-1a**	100%	1870.4 ± 2298	1029.8	30.9 _ 14021.9
**IL-1ra**	100%	2009.6 ± 2027.7	1459.9	55.8 _ 13147.7
**IL-8 (CXCL8)**	100%	315.5 ± 355.1	213.2	11.4 _ 2114.2
**IL-18**	100%	93.2 ± 119.7	50.6	3.4 _ 866.5
**MCP-1 (CCL2)**	100%	479.9 ± 634.2	325	30 _ 6405.4
**GRO-a (CXCL1)**	98%	109 ± 163.4	69.8	1.3 _ 1418.7
**VEGF-A**	98%	148.8 ± 161.1	97.2	2.6 _ 1154.1
**G-CSF**	97%	103.9 ± 109.5	69.2	2.6 _ 708
**IP-10 (CXCL10)**	95.5%	149.2 ± 323.4	43.7	1.5 _ 2332.8
**IL-1B**	93%	164.3 ± 342.7	39.5	1.5 _ 2647.5
**IL-6**	93%	8.3 ± 13.7	3.8	0.6 _ 96.6
**TGF-a**	87%	7.7 ± 7	5.4	1.3 _ 32.8
**Eotaxin-1 (CCL11)**	85%	14.6 ± 13.1	11.6	3 _ 94.5
**CD40L**	70%	77.4 ± 88.8	55.8	9.3 _ 693.6
**IL-10**	63%	9.4 ± 21.9	4	2.6 _ 268.9
**IL-9**	62%	8.9 ± 8.3	8.4	0.6 _ 42.7
**IL-12p40**	57%	23 ± 50.7	8.1	6.3 _ 478.7
**RANTES (CCL5)**	57%	3.7 ± 5.9	1.5	1.3 _ 66.9
**Fractalkine (CX3CL1)**	51%	70.9 ± 72.7	31.3	28.2 _ 481.7
**MDC (CCL22)**	53%	1.7 ± 2	0.7	0.6 _ 12.9
**IFN-g**	53%	4.4 ± 6.3	1.4	1.3 _ 37.3
**FGF-2**	48%	56.4 ± 66.4	26	26 _ 542.2
**Flt-3L**	47.5%	1.6 ± 1.5	1	1 _ 10.6
**IL-15**	46%	4.4 ± 2.7	3	3 _ 25.4
**TNF-b**	45%	6.3 ± 9.7	1.6	1.6 _ 92.7
**MCP-3 (CCL7)**	44%	12.5 ± 8.6	8	8 _ 84.5
**TNF-a**	41%	12.7 ± 20.3	6.4	6.4 _ 218.1
**IL-12p70**	41%	5.6 ± 6.1	3	3 _ 41.3
**MIP-1b (CCL4)**	35%	2.1 ± 3.8	0.4	0.4 _ 25.1
**IL-13**	33%	13.4 ± 22.4	6.4	6.4 _ 238
**IL-2**	31%	1.2 ± 1.3	0.6	0.4 _ 9.9
**IFN-a2**	30%	13.1 ± 21.7	8	7.5 _ 262.9
**IL-17A**	26%	3.1 ± 5.5	1.3	1.3 _ 56.6
**IL-4**	20%	0.8 ± 0.7	0.6	0.6 _ 6.5
**IL-5**	20%	1 ± 1.3	0.6	0.6 _ 10.3

**Table 2:** Binary variables (demographics, medical records, socioeconomic status, birth outcome and oral health information) for two clusters among 181 pregnant women

Binary Variables	Low n = 127	High n = 54	
	proportion	proportion	Chi-sq test p-value
Demographics			
Age > 30	26.77%	38.89%	0.148
Black	54.33%	50.00%	0.710
Hispanic	11.81%	12.96%	1
Medical Record			
Yeast Infection	22.83%	14.81%	0.307
Antibiotics	3.15%	1.85%	1
Antifungal	6.30%	9.26%	0.696
Inhaler Use	9.45%	7.41%	0.876
Diabetes	4.72%	11.11%	0.21
Asthma	12.60%	12.96%	1
Emotional condition	31.50%	38.89%	0.429
Depression score > 12	54.33%	50.00%	0.915
Hypertension	12.60%	16.67%	0.624
Smoking	16.54%	11.11%	0.478
Socioeconomic Status			
Employed	51.18%	50.00%	1
Married	17.32%	33.33%	< 0.05
Birth Outcome			
Low birth weight	2.36%	7.41%	0.234
Oral Health			
Brush Twice Daily	62.99%	70.37%	0.434
Salivary *Candida* (Yes)	56.69%	38.89%	< 0.05
Salivary *S. mutans* (Yes)	95.28%	83.33%	< 0.05
Plaque *Candida* (Yes)	43.31%	16.67%	< 0.001
Plaque *S. mutans* (Yes)	89.76%	66.67%	< 0.001

**Table 3:** Continuous variables (demographics, birth outcome, oral health information, and hormones levels) for two clusters among 181 pregnant women

Continuous Variables	Low n = 127	High n = 54	
	mean ± sd	mean ± sd	p-value
Demographics			
Age (year)	27.22 ± 4.92	28.59 ± 6.62	0.268
Gestational Week	33.69 ± 5.73	33.31 ± 3.47	0.986
Birth Outcome			
Infant Birth weight	3293.34 ± 423.24	3234.96 ± 654.97	0.859
Oral Health			
PI	1.64 ± 0.61	1.65 ± 0.59	0.914
DT	2.87 ± 3.41	2.43 ± 4.21	0.170
MT	0.88 ± 1.5	1.56 ± 4.23	0.771
FT	3.2 ± 3.55	2.5 ± 3.14	0.258
DMFT	6.94 ± 4.97	6.39 ± 6.02	0.218
DS	4.61 ± 7.72	2.96 ± 6.77	0.055
MS	4.29 ± 6.89	7.41 ± 19.53	0.809
FS	5.69 ± 7.31	3.63 ± 6.69	0.053
DMFS	14.57 ± 14.03	14 ± 20.77	0.110
ICDAS	3.44 ± 2.49	3.47 ± 2.5	0.899
Salivary *C. albicans* Ln (CFU/ml× 10^2^)	3.64 ± 3.73	2.28 ± 3.32	< 0.05
Salivary *S. mutans* Ln (CFU/ml× 10^5^)	11.9 ± 3.3	10.65 ± 5.14	0.498
Plaque *C. albicans* Ln (CFU/ml)	3.51 ± 4.45	0.91 ± 2.49	< 0.0001
Plaque *S. mutans* Ln (CFU/ml)	11.62 ± 4.88	8.59 ± 6.48	< 0.05
Hormones Levels			
Cortisol Ln (pg/mL)	1.8 ± 0.76	1.34 ± 0.88	< 0.0001
Estradiol Ln (pg/mL)	6.16 ± 1.66	5.18 ± 2.38	< 0.001
Progesterone Ln (pg/mL)	8.98 ± 1.77	8.24 ± 2.43	0.133
Testosterone Ln (pg/mL)	5.75 ± 2.65	3.89 ± 3.37	< 0.001
T3 Ln (pg/mL)	5.13 ± 2.65	3.42 ± 3.39	< 0.001
T4 Ln (pg/mL)	3.74 ± 3.43	2.35 ± 3.3	< 0.05

PI: Plaque index. DT: decayed teeth. MT: Missing teeth. FT: Filled teeth. DMFT: Decayed, Missing, and Filled Teeth. DS: decayed surface. MS: Missing surface. FS: Filled surface. DMFS: Decayed, Missing, and Filled surface. ICDAS: International Caries Detection and Assessment System.

**Table 4:** Logistic Regression model with LASSO penalty

Variables	coefficients
Demographics	
Age > 30	.
Black	.
Hispanic	.
Medical Record	
Yeast Infection	.
Antifungal	.
Asthma	.
Emotional condition	.
Hypertension	.
Inhaler use	.
Diabetes	0.160
Smoking	.
Socioeconomic Status	
Employed	.
Married	0.196
Birth Outcome	
Adverse Birth Outcome	.
Low birth weight	0.109
Hormones Levels	
Cortisol Ln (pg/mL)	−0.180
Estradiol Ln (pg/mL)	.
Progesterone Ln (pg/mL)	.
Testosterone Ln (pg/mL)	−0.065
T3 Ln (pg/mL)	.
T4 Ln (pg/mL)	.
Oral Health	
PI	.
DT	.
BrushTwiceDaily	.
Salivary *Candida* (Yes)	.
Salivary *S. mutans* (Yes)	.
Plaque *Candida* (Yes)	−0.574
Plaque *S. mutans* (Yes)	−0.763

**Table 5:** Post-selection Inference (Debiased LASSO)

Variable	Coefficients	p-values	CI lower	CI upper	sd
Married	0.53	0.46	−2.02	1.31	0.41
Diabetes	1.07	0.63	−7.23	1.81	0.66
Low birth weight	1.25	0.79	−18.71	1.82	0.85
Plaque *Candida* (Yes)	−1.19	0.02	−2.08	−0.3	0.43
Plaque *S. mutans* (Yes)	−1.01	0.03	−1.67	−0.12	0.38
Testosterone Ln (pg/mL)	−0.09	0.29	−0.35	0.19	0.08
Cortisol Ln (pg/mL)	−0.37	0.37	−1.1	1	0.28

## Data Availability

A complete record of all the data collected or analyzed during this study is provided in this paper. The corresponding author can be contacted for further information.
